# Identification of Germinal Neurofibromin Hotspots

**DOI:** 10.3390/biomedicines10082044

**Published:** 2022-08-21

**Authors:** Sergio Lois, Juan Báez-Flores, María Isidoro-García, Jesus Lacal, Juan Carlos Triviño

**Affiliations:** 1 Department of Bioinformatics, Sistemas Genómicos, 46980 Paterna, Spain; 2 Institute for Biomedical Research of Salamanca (IBSAL), 37007 Salamanca, Spain; 3 Laboratory of Functional Genetics of Rare Diseases, Department of Microbiology and Genetics, University of Salamanca, 37007 Salamanca, Spain; 4 Department of Clinical Biochemistry, University Hospital of Salamanca, 37007 Salamanca, Spain; 5 Department of Medicine, University of Salamanca, 37007 Salamanca, Spain; 6 Asthma, Allergic and Adverse Reactions (ARADyAL) Network for Cooperative Research in Health of Instituto de Salud Carlos III, 37007 Salamanca, Spain

**Keywords:** neurofibromin, *NF1*, neurofibromatosis type 1, germinal variants, pathogenic variants

## Abstract

Neurofibromin is engaged in many cellular processes and when the proper protein functioning is impaired, it causes neurofibromatosis type 1 (*NF1*), one of the most common inherited neurological disorders. Recent advances in sequencing and screening of the *NF1* gene have increased the number of detected variants. However, the correlation of these variants with the clinic remains poorly understood. In this study, we analyzed 4610 germinal *NF1* variants annotated in ClinVar and determined on exon level the mutational spectrum and potential pathogenic regions. Then, a binomial and sliding windows test using 783 benign and 938 pathogenic *NF1* variants were analyzed against functional and structural regions of neurofibromin. The distribution of synonymous, missense, and frameshift variants are statistically significant in certain regions of neurofibromin suggesting that the type of variant and its associated phenotype may depend on protein disorder. Indeed, there is a negative correlation between the pathogenic fraction prediction and the disorder data, suggesting that the higher an intrinsically disordered region is, the lower the pathogenic fraction is and vice versa. Most pathogenic variants are associated to *NF1* and our analysis suggests that GRD, CSRD, TBD, and Armadillo1 domains are hotspots in neurofibromin. Knowledge about *NF1* genotype–phenotype correlations can provide prognostic guidance and aid in organ-specific surveillance.

## 1. Introduction

Neurofibromatosis type 1 (*NF1*) (OMIM#162200) is one of the most common autosomal dominant diseases of the nervous system, with an estimated incidence of approximately 1 in 3000 births [1] and it has phenotypic overlap with other RASopathies [2]. The *NF1* gene, located in the pericentromeric region of chromosome 17q11.2, spans 350 kb of genomic DNA which contains 60 exons that encode a transcript in the direction from the centromere to the telomere, giving rise to a mRNA of 11 to 13 kb with an open reading frame of 9 kb [3,4]. *NF1* occurs as a result of a germline mutation in 1 of the 2 alleles of the *NF1* gene. However, somatic loss of function in the second allele is required for tumor formation [5]. The allele responsible for *NF1* is inherited in an autosomal dominant pattern and the *NF1* phenotype is complex due to variable expressivity and pleiotropy. The phenotypic complexity of *NF1* is likely multifactorial, including epigenetic phenomena, stochastic events, and heritable elements such as genetic modifiers [6].

The *NF1* gene encodes neurofibromin, a Ras-GTPase activating protein (GAP) that promotes the conversion of an active Ras-GTP bound form to an inactivate Ras-GDP form and functions to negatively regulate the activity of Ras/MAPK [7], Raf/MEK/ERK [8], PI3K/Akt/mTOR [9], Rho/ROCK/LIMK2/cofilin [10], PKA-Ena/VASP [11], and cAMP/PKA pathways [12], which when left unchecked, results in a cellular overproliferation and tumor formation [13]. Neurofibromin is ubiquitously expressed and is present in most tissues acting as a tumor suppressor protein but most highly in particularly high levels in the central nervous system, including Schwann cells along peripheral nerve trunks, glial cells, oligodendrocytes, astrocytes leukocytes, adrenal medulla, and neurons, which partially explains the predilection for peripheral nerve sheath tumors and gliomas [14]. Neurofibromin has several predicted functional domains and regions including the PKC domain (protein kinase C, 97–243 residues), CSRD (cysteine-serine rich domain, 543–909 residues), TBD (tubulin binding domain, residues 1095–1197), GRD (GAP-related domain, residues 1198–1530), Poly-Ser (1352–1355 residues), Sec14-PH (also known as CRAL-TRIO lipid binding domain, residues 1560–1816), Armadillo-type fold superfamily domains (residues 1849–1886, 1920–1984 and 2200–2571), the CTD (C-terminal domain, residues 2260–2818) including NLS (bipartite nuclear localization signal domain, 2534–2550 residues), and SBR (syndecan-binding regions, residues 1357–1473 and 2619–2719). Neurofibromin is known to associate with a large number of proteins, including but not limited to FAF2/ETEA [15], HTR6 [16], SPRED1 [17], tubulin [18], kinesin [19], protein kinase A [20], protein kinase C [21], syndecan [3], caveolin [22], cytokeratin intermediate filaments [23], nuclear PML-bodies [24], p97/VCP [25], and the amyloid precursor protein [26]. Although the biological significance of these protein–protein interactions is largely unknown [5], the diversity of protein associations does emphasize the point that neurofibromin is likely to have many functions other than merely functioning as a GAP protein [5]. 

Neurofibromin acts as a tumor suppressor protein and alterations in the *NF1* gene are related to neurofibromatosis type 1, which is the first human condition mapped to the RAS pathway and has been shown to originate from germline mutations [27]. The main clinical features associated with *NF1* include café-au-lait macules (CALMs), 99% of patients with *NF1* have fulfilled this criteria by age 1 [28], skinfold freckling (SF) [29], Lisch nodules (LN) [30], and optic pathway glioma (OPG) [31]. Moreover, neurofibromas (NFs), cutaneous neurofibromas (cNF), and plexiform neurofibromas (pNF) are the most prevalent benign peripheral nerve sheath tumor, although the plexiform type is pathognomonic for *NF1* and carries an increased risk of malignant transformation (MPNSTs) affecting around 50% of patients with *NF1* [32]. MPNSTs are the leading cause of death (around 45%) in this population [33], with a 5-year survival rate of 15% to 50% [34]. Moreover, some individuals develop other symptoms such as skeletal abnormalities, vascular injuries, learning disabilities, attention deficit, increased susceptibility to autism, and social and behavioral problems [35]. Hematopoietic neoplasms are also associated with clinical manifestations in patients with *NF1*, most often leukemia infantile myelomonocytic syndromes myelodysplastic [36] or the presence of pheochromocytoma [37]. *NF1* has been shown to be an essential gene for embryonic development, and mice lacking a functional gene die in utero due to generalized pneumothorax and cardiovascular defects [38]. Additionally, the alterations in the *NF1* gene are related to several human highly aggressive malignancies diseases including *NF1*, glioblastoma [39], melanoma [28], ovarian carcinoma [40], lung cancer [41], cholangiocarcinoma [42], breast cancer [43], lymphoblastic leukemia [28], and other types of tumors [44].

*NF1* genotype–phenotype correlations lead to increasing demand to pursue genetic testing to understand the type of pathogenic mutation and permit more precise interventions. Genotype–phenotype correlations in *NF1* are not well known although some have been reported [45]. In addition, recent genotype–phenotype correlations studies suggest that *NF1* may be more relevant in tumor initiation and progression than previously thought [46]. Despite all these studies, the association between the risk of developing *NF1* and specific germline *NF1* mutations is still debated, and further studies are needed. In this manuscript, we analyzed the distribution of 4610 germinal variants of the *NF1* gene annotated in ClinVar database. Using this strategy, we identified potential functional neurofibromin hotspots enriched in pathological variants, their functional, and phenotype consequence. Additionally, considering data integration of functional and structural neurofibromin variables, we suggest a new regression model that explains the specific *NF1* pathological hotspot distribution.

## 2. Materials and Methods

### 2.1. Neurofibromin Residues, Regions, and Domains Defined in This Study

Neurofibromin functional domains were obtained from Uniprot database [47], P21359; UniProt, as well as from Interpro [48], Prosite [49], and previous published papers [50,51].

### 2.2. Analysis of NF1 Variants from Clinvar

*NF1* variants used in this study were obtained from ClinVar database, release of February of 2020 [52]. For the study of the *NF1* germinal mutational spectrum and potential pathogenicity, 2365 out of 4610 variants were selected based on their exonic position, including variants that affected the splicing sites of the introns. For the remaining studies of the manuscript, 1721 out of 4610 variants were studied and classified in two categories, benign if the clinical significance described in ClinVar was benign or likely benign, and pathological if the clinical significance described in ClinVar was pathological or likely pathological. The effect of the variants was obtained using variant effect predictor [53] with the canonical (isoform II) amino acid sequence of neurofibromin.

### 2.3. Sliding Windows Test

A sliding window analysis was used to plot test statistics with a sliding window at 100 amino acids length and sliding of 25 amino acids along the sequence of neurofibromin. Neither the 3D structure of neurofibromin nor a predicted 3D model that meets the energy requirements is available. Therefore, to provide the structural information we used the predicted secondary structure of neurofibromin, including alpha helix, beta lamina, “Coil”, and disorder information using RaptorX algorithm [54]. Additionally, we displayed GERP conservation scores for each window [55].

### 2.4. Identification of Pathogenic Variant Enriched Regions across Neurofibromin

The enrichment of pathogenic variants proportion for each of the 110 windows previously stablished in neurofibromin was performed using a binomial test. The neurofibromin global fraction between pathogenic and benign variants was fixed in 0.52. The *p*-values, based on a binomial test, were obtained from such analyses for each domain of neurofibromin.

### 2.5. Models Performance

For the pathological variants, the fractions prediction was obtained using a lineal regression model which was generated using functional protein domain and structural data. The correlations between empirical pathological variants fractions and predicted values were evaluated using the Pearson correlation coefficient.

## 3. Results

### 3.1. NF1 Germinal Mutational Spectrum and Potential Pathogenicity

**Figure 1:**
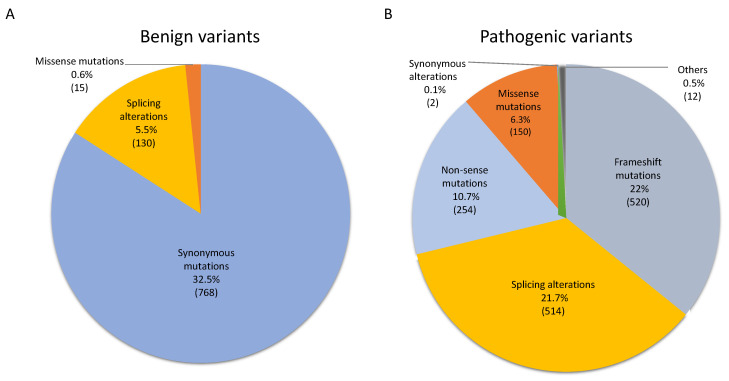
*NF1* germinal mutational spectrum and potential pathogenicity. A total of 2365 variants were classified in different categories including missense 165 (7%), 254 non-sense (10%), 520 frameshift (22%), 644 splicing alterations (27%), 770 synonymous (32%), and 12 are annotated as others (2%). They were also classified as (**A**) benign (913, 39%) or (**B**) pathogenic (1452, 61%) based on the annotation as reported in ClinVar.

Based on the distribution of the elevated number of pathological *NF1* variants found in human samples and previous pathological genotype–phenotype correlations as mentioned in the introduction, we hypothesized that some regions of neurofibromin may be more determining than others, when they are mutated and depending on the type of mutation, to present a clinical manifestation. To investigate this hypothesis, we analyzed a total of 4610 germinal *NF1* variants found in the ClinVar database (M&M). Out of the 4610 germinal variants, we eliminated those at the intron level that were not affecting the splicing positions, resulting in 2365 variants. These variants were classified in different categories including missense 165 (7%), 254 non-sense (10%), 520 frameshift (22%), 644 splicing alterations (27%), 770 synonymous (32%), and 12 are annotated as others (2%) (Figure 1). They were also classified as benign (913, 39%) (Figure 1A) or pathogenic (1452, 61%) (Figure 1B) as reported in ClinVar. As expected, most benign variants are found in synonymous mutations (768, 32.5%), then splicing alterations (130, 5.5%) and missense (15, 0.6%) (Figure 1A). On the other hand, pathogenic mutations are predominantly found in frameshift (520, 22%), splicing alterations (514, 21.7%), non-sense (254, 10.7%), missense mutations (150, 6.3%), synonymous (2, 0.1%) and others (12, 0.5%) (Figure 1B). 

### 3.2. Neurofibromin CSRD, GRD, Armadillo1, and the TBD Are Hotspots

**Figure 2:**
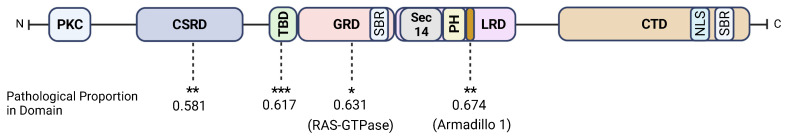
Pathological proportion in neurofibromin domains. Based on the 0.52 threshold obtained as reported in M&M and using *p*-values up to 0.1 as the cut-off for significance, our analysis identified four hotspots. The RAS-GTPase domain *p* < 0.01 * (0.003), the CSRD domain and the Armadillo1 domain with *p* > 0.01 and *p* < 0.05 **, and the TBD with a *p* > 0.05 and < 0.1 *** (0.078).

In order to identify potential hotspots in *NF1*, 1721 out of 4610 germinal variants from ClinVar were selected for further studies using a binomial method (M&M). Excluded variants correspond to those annotated as conflicting variants in ClinVar and those that do not affect the coding region of *NF1*. On the other hand, those annotated as benign or likely-benign (783 final variants) and those annotated as pathogenic or likely-pathogenic (938 final variants) were selected to do a binomial test against to 18 functional and structural regions of neurofibromin (Figure 2). Out of the 1721 germinal variants, 1236 are located among the 18 regions, including 663 pathogenic variants. Based on these numbers, the test results establish a full-length pathological proportion of 0.52 across neurofibromin, meaning that values over 0.52 are statistically significant pathogenic. Based on that threshold and using a *p*-value of 0.05 as the cut-off for significance, our analysis identifies three regions within neurofibromin that are statistically significantly higher than the threshold, suggesting them as hotspots. These regions are the RAS-GTPase domain (*p* < 0.003), the CSRD (*p* < 0.02), and the Armadillo1 (*p* < 0.03) **(**Figure 2). These three regions harbor 43.5% of the 938 pathogenic *NF1* variants used in this study. The TBD has a pathological proportion of 0.62, which is above the pathological proportion of 0.52, with a *p* > 0.05 and < 0.1, suggesting that this domain may be also a hotspot (Figure 2).

In order to determine the germinal mutational spectrum of *NF1* hotspots, an enrichment analysis of the type of pathological variants within neurofibromin, using the same binomial strategy was carried out (Table S1). Focusing on the pathogenic variants, our results indicate that missense variants are the most abundant in the CSRD (35 out of 35), the RAS-GTPase (31 out of 32), and TBD (9 out of 15) domains, with a *p*-value of 5 × 10^−6^, 5 × 10^−5^, and 5 × 10^−3^, respectively (Table S1). Two nonsense variants, with a *p*-value of 0.002, and five missense variants, with a *p*-value of 0.055, are found in the Armadillo1 domain (Table S1). Therefore, the enrichment analyses strongly support the existence of hotspot regions within neurofibromin. 

### 3.3. Neurofibromin CSRD and GRD Domains Are Further Validated as Hotspots Based on the Sliding Window Test

**Figure 3:**
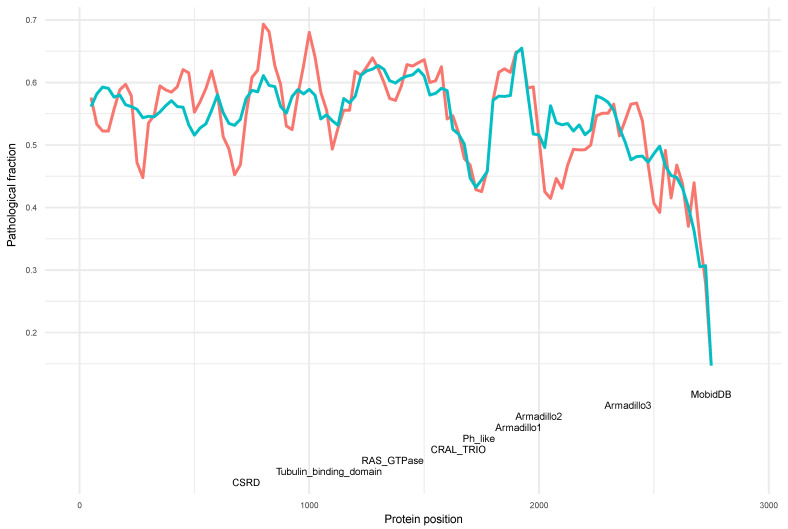
Estimated pathological fraction across neurofibromin full length protein sequence. The continuous blue line corresponds to the predicted pathological fraction using the global model, whereas the red line indicates the proportion of pathological variants of each window. Neurofibromin domains are represented by different colors.

To provide further evidence of the identified hotspots in neurofibromin, we performed a sliding window test (M&M). The neurofibromin protein sequence was divided in frames of 100 amino acids and with sliding of 25, resulting in 110 different windows. Using this strategy, the local pathological variant proportion and statistical significance was calculated for each window (Figure 3). The hotspot regions were identified using a *p*-value threshold of 0.1 as the cut-off for significance. The results show a high degree of concordance with the binomial method applied in the previous test. The RAS-GTPase domain and CSRD present 5 and 3 windows, respectively, with significantly high pathological proportion. Additionally, the Armadillo1 protein domain presents its two windows as significant and, in contrast, the TBD does not have any (Figure 3).

The windows of the CSRD domain present a very uneven proportion with the first half of the domain (residues 550 to 725) containing a low/medium pathological proportion with a mean of 0.53, whereas the last three windows in the second half (residues 750 to 900) have a mean value of 0.66. This result suggests that the global high pathological proportion of this domain is mainly concentrated in the last 3 windows. On the other hand, the RAS-GTPase domain presents two regions in both edges of the domain (residues 1175 to 1350 and 1400 to 1550) with the highest pathological proportion (Figure 3). The two windows of the Armadillo1 have significant pathological proportion, suggesting that this domain presents more compact structural parameters than larger domains (Figure 3). In addition to the aforementioned domains, our results show that the region between the CSRD and the TBD, from 950 to 1050 residues, has the highest (0.68) pathological proportion and with *p*-value of 0.009, suggesting that it may has an important structural role in neurofibromin stability. 

### 3.4. Distribution of Variants along the Neurofibromin Protein

**Figure 4:**
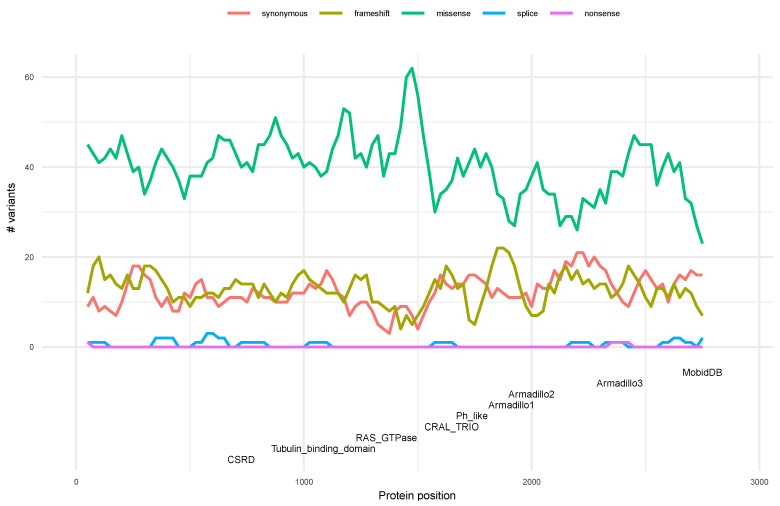
Variants distribution in neurofibromin. A total of 4610 variants annotated in ClinVar were used for the study. Different types of variants are represented by continuous lines using different colors. Neurofibromin protein domains are defined at the bottom.

Using the sliding windows strategy, it is possible to analyze the statistical distribution of the type of variants along neurofibromin (Figure 4).

In order to carry out this analysis, all 4610 variants were selected for the study. Our results show that the distribution of the synonymous, missense, and frameshift variants, using a *p*-value threshold of 0.05, is statistically significant in certain regions of neurofibromin (Table S2). The synonymous variants are statistically significant in the C terminal region of the neurofibromin (residue 2050 to 2375) in the Armadillo3 domain. Regarding the missense variants, 9 out of 12 windows of the RAS-GTPase domain (residue 1250 to 1550) present a high statistically significance, as well as in 2 windows of the CSRD (residue 575 to 900) (Figure 4). On the other hand, the number of splice and nonsense variants by window are very low (less than 5 variants) making it impossible to apply the binomial test.

The frameshift variants present a greater heterogeneity in protein distribution. However, they are concentrated in two regions, the Armadillo1 domain (residue 1800 to 1975) with a mean of *p*-value of 0.003 and the CRAL-TRIO (residue 1575 to 1675) with a mean of *p*-value of 0.002. Interestingly, the windows with significant frameshift variants present a higher percentage of disorder than windows with missense variants (Wilcoxon *p*-value 0.0009). This result suggests that the impact of the type of variant and its associated phenotype may depend on the grade of disorder of the region. This relationship could help to improve the understanding of the pathogenicity of *NF1* variants. More and deep analysis should be carried out to corroborate this hypothesis.

### 3.5. Phenotype Distribution of Pathogenic Variants along Neurofibromin

**Figure 5:**
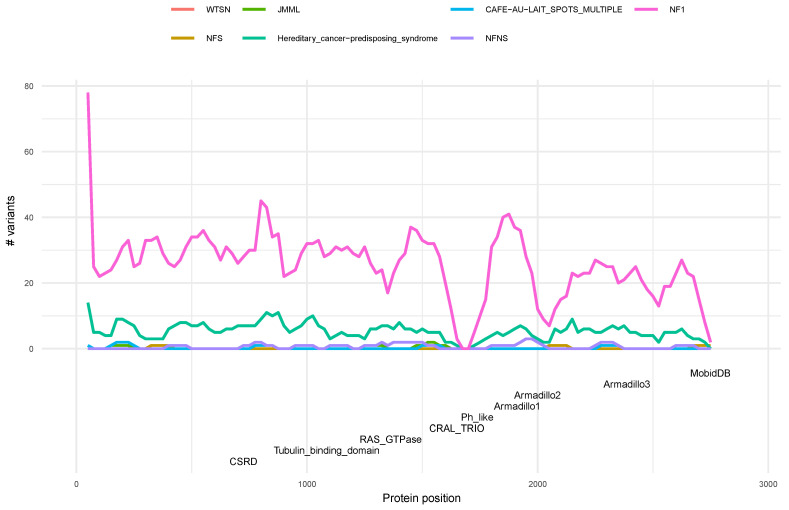
Distribution of phenotypes across neurofibromin based on the sliding window test. The continuous line and colors indicate the distribution of variants according to their phenotype as annotated in ClinVar. Neurofibromin protein domains are defined at the bottom.

Using the same windows strategy as above, a phenotype distribution along neurofibromin was carried out. Based on ClinVar annotations, although a total of seven syndromes are associated to *NF1* variants, only two phenotypes could be statistically evaluated the “*NF1* type1” and the “hereditary_cancer-predisposing_syndrome” (Figure 5). The rest of the phenotypes presented a low number of variants by window and therefore were excluded from the analysis. Our results show that the RAS-GTPase domain presents enrichment with 3 windows (residue 1250 to 1400) with “Hereditary_cancer-predisposing_syndrome” as the significant phenotype, whereas the same domain but in a different position (residues 1375 to 1475) presents significance in “*NF1* type1” (Table S2). This analysis presents some limitations due to the number of variants studied, making it necessary to perform new analysis using future releases of the databases with deeper variants information. Despite the limitations, our results suggest that the localization of the variants within a particular domain is associated to a specific phenotype.

### 3.6. Neurofibromin Functional and Structural Relationships within a Pathological Context

The windows analysis also allowed us to obtain deeper functional and structural relationships within a pathological context. In particular, residues 750 to 875 within the CSRD have the highest pathological proportion (0.69; *p*-value of 0.002) across neurofibromin, whereas residues 575 to 725 within the CSRD have the lowest pathological proportion (0.49; *p*-value of 0.82) (Figure 3, Table S2). Therefore, according to these results neurofibromin windows are far from being homogeneous; instead, they present valleys and peaks indicating that some regions are more pathogenic than others, even within a particular domain. 

To establish a pathogenic correlation between functional and structural parameters, the results from the sliding windows test were correlated with some structural parameters of neurofibromin (M&M). We analyzed the "% disorder area” for each window of neurofibromin with respect to the pathological proportion. The results show that there is a negative correlation between the pathogenic fraction prediction and the % disorder data (Pearson correlation of −0.65, *p*-value of 8.27 × 10^−15^), suggesting that the higher an intrinsically disordered region is within a particular area, the lower the pathogenic fraction is, and the other way around. Additionally, other structural variables including the percentage of Helix and Barrier structure, B factor, and Coil irregular, were significantly correlated with the pathological fraction, although with a lower correlation than the structural disorder estimation. The most interesting structural variables were the percentage of Helix and Barrier structure, with a Pearson correlation of 0.33 and 0.32, respectively. 

To integrate the above functional and structural information, a lineal model was generated as described in M&M. Briefly, protein domain, structural disorder area estimation, helix context, and evolutionary conservation were integrated into the model for pathological fraction prediction. The resulting model presents a high degree of correlation (Pearson correlation of 0.84) and a *p*-value < 1 × 10^−16^ with the proportion of pathological variants (Figure S1). These results indicate that the hotspots of neurofibromin can be explained as a sum of functional protein domain and some structural protein parameter. The windows with a specific functional role, low disorder area, high percentage of helix and high evaluative conservation present higher probability of being a hotspot. 

## 4. Discussion

Increased efforts towards the identification of additional clinically relevant genotype–phenotype correlations in patients carrying *NF1* mutations are needed. Therefore, in this study, we aimed to identify on exon level potential hotspots in neurofibromin in the context of germinal variants found in human samples annotated in ClinVar database. To investigate precise genotype–phenotype associations across the *NF1* locus, cohorts bigger than 307 patients should be used [56]. Out of 2365 germinal *NF1* variants in ClinVar, and as expected, some of these mutations were synonymous (32%) and did not affect the protein sequence of neurofibromin, whereas the remaining variants did (68%). The top three most abundant type of germinal variants include splicing alterations (27%), frameshift (22%), and non-sense (10%), followed by missense (7%) and other types (2%). From these data, one may infer that splicing, frameshift, and non-sense mutations are predominant in *NF1* related syndromes. Furthermore, these alterations would cause greater phenotypic alterations than missense alterations due to its greater impact on the protein structure. As previously noted in 2018, out of 3786 *NF1* variants submitted to ClinVar [52], 1594 (43%) were classified as variants of uncertain clinical significance (VUS), which are highly problematic since they can cause confusion among patients and professionals [57]. Therefore, precise classification of variants is key for a proper clinical management.

When we analyzed the statistical distribution of the type of variants along neurofibromin, we found that some are significantly concentrated in some regions. Synonymous variants are more abundant in the CTD, missense variants in the GRD, and the CSRD, whereas frameshift variants are concentrated in the Armadillo1 domain and the CRAL-TRIO. Interestingly, regions with significant frameshift variants present a greater structural disorder percentage than regions with missense variants. This suggests that mutations that fall into areas of greater structural disorder in neurofibromin must have a greater functional impact on the protein (such as frame shifts vs missense mutations) for a clinical phenotype to occur. This relationship could be interesting in the categorization of variants of uncertain meaning considering the structural region and its protein impact [58]. However, more deep studies based in broad groups of genes must be done to corroborate this relationship. 

Out of the 4610 variants annotated in ClinVar, 783 (17%) are predicted benign, mostly because they are synonymous mutations. Despite there being evidence that synonymous mutations frequently contribute to human cancer [59], such studies have not been performed in *NF1* yet, perhaps assuming that synonymous mutations may not alter neurofibromin function. To the best of our knowledge, no germline *NF1* silent mutations have been associated to cancer or neurofibromatosis type 1 so far. On the other hand, 938 (20%) variants are predicted to be pathogenic. The remaining variants (2889, 63%) were excluded from this study based on their uncertain phenotypic classification. Indeed, since the discovery of *NF1*, due to its large size and the heterogeneity of mutation types and positions, it has been difficult to predict and identify the impact of most mutations. Furthermore, phenotypic differences in *NF1* patients are more likely to be caused by mechanisms such as “a second hit”, modifying genes that are unlinked to the *NF1* locus, epigenetic alterations, or other environmental factors [60]. Therefore, more translational studies are needed to pinpoint the impact of variants in *NF1* etiology. 

*NF1*-related pathogenicity may correlate with the location of the mutation so we investigated for potential hotspots in neurofibromin and identified three hotspots including the CSRD, the RAS-GTPase domain, and the Armadillo1. These three regions harbor 43.5% (408/938) of the pathogenic *NF1* variants used in this study. Both the binomial and sliding windows tests carried out in this study were concordant with each other, strongly supporting the existence of these three hotspots in neurofibromin in the onset of germinal mutations. The analysis of the *NF1* gene is challenging and previous studies were not able to prove the existence of hotspots areas in *NF1*, suggesting the lack of mutation hotspots [61]. However, a limited number of mutational hotspots were identified in the coding sequence of *NF1*: a recurrent missense mutation Y489C (A1466G) associated with aberrant splicing in exon 10b [62], codons 844-848 in the CSRD [63], 992 [63] and 1149 in the TBD [63], 1276 and 1423 in the GRD [63], 1809 in the PH [63], R1947X (C5839T) in exon 31 [64], and the 4-bp region between nucleotides 6789 and 6792 in exon 37 [65]. In addition, apart from exon 37, mutations distributed along the *NF1* gene in *NF1* patients showed other seven exons/flanking introns in which mutations are represented more often (4b, 7, 10b, 13, 15, 20, and 29), where 77 of the 189 identified mutations are located (41%), although they represent only 16% of the coding region [66]. Locally, the GRD region has been proposed to be a hotspot for missense mutations [67]. All the above mutations were associated with *NF1* phenotypes. Interestingly, previous hotspots 844–848, 992, 1276, and 1423 do correlate with our findings as hotspot areas. Our results strongly support the existence of hotspot areas within neurofibromin CSRD, GRD, and Armadillo 1 domains. 

Similarly, we estimated the phenotype distribution of pathogenic variants along neurofibromin. Interestingly, the RAS-GTPase domain is enriched in “hereditary_cancer-predisposing_syndrome” and “*NF1* type 1”, being the only domain showing a clear genotype–phenotype correlation. The limitations encountered due to the reduced number of variants annotated may be overcome with more analyses using future releases of the databases containing deeper variants information. Regarding the CSRD domain, previous studies suggested that patients harboring mutations in this domain had a higher risk of developing optic pathway glioma OPG than patients with mutations in other regions [4], however, further studies are still needed to confirm associations between *NF1* genotype and OPG phenotype. On the other hand, the risk of developing a glioma was not associated with particular patterns of *NF1* gene mutations in the patient’s germline DNA. The *NF1* mutations observed in germline DNA were typically truncating and frameshift and did not cluster into specific domains of neurofibromin. 

We also analyzed the possible correlation and statistical significance between the proportion of pathological variants by windows defined in the *NF1* gene and some structural and functional parameters. In this context, the results show that the percentage of disorder and other secondary structures from the protein presented a high level of correlation and statistical significance. The negative correlation between percentage disorder and the pathological proportion and the positive correlation of other secondary structure such as percentage helix could indicate that the level of structure of a specific region could present some influence in the hotspot definition. Functional variables were only evaluated if the region belonged to a functional domain defined in the databases. 

Finally, the integration of the neurofibromin structural and functional variables was evaluated. The integration of protein structure information including the percentage of disorder and alpha helix, evolutionary conservation of the region, and whether or not a certain region belonged to a functional domain was used to define a region as a hotspot. This integration allows the understanding of the pathogenicity mechanisms of *NF1* as a sum of structural and functional variables and opens the possibility for the use of this information in clinical interpretation of novel or complex variants. Interestingly, our data present a high grade of correlation and may be even extrapolated to other genes involved in diseases. We expect that future deeper analysis using more structural and functional parameters allow to deepen in these pathological mechanisms and its possible relationships with useful clinical information.

## Data Availability

The data that support the findings of this study are available from the corresponding author upon reasonable request. We have been granted a license to use the BioRender content in this manuscript: JD247JS4HS.

## Supplementary Materials

The following supporting information can be downloaded at: https://www.mdpi.com/article/10.3390/biomedicines10082044/s1, Figure S1: A lineal model with a high degree of correlation; Table S1: Germinal variant type analysis in neurofibromin domains; Table S2: Sliding windows analysis.
