# Supplementary figures and images for: Identification of Germinal Neurofibromin Hotspots
Source: Biomedicines. 2022 Aug 21;10(8):2044. doi: 10.3390/biomedicines10082044 (PMC9405573; doi:10.3390/biomedicines10082044)

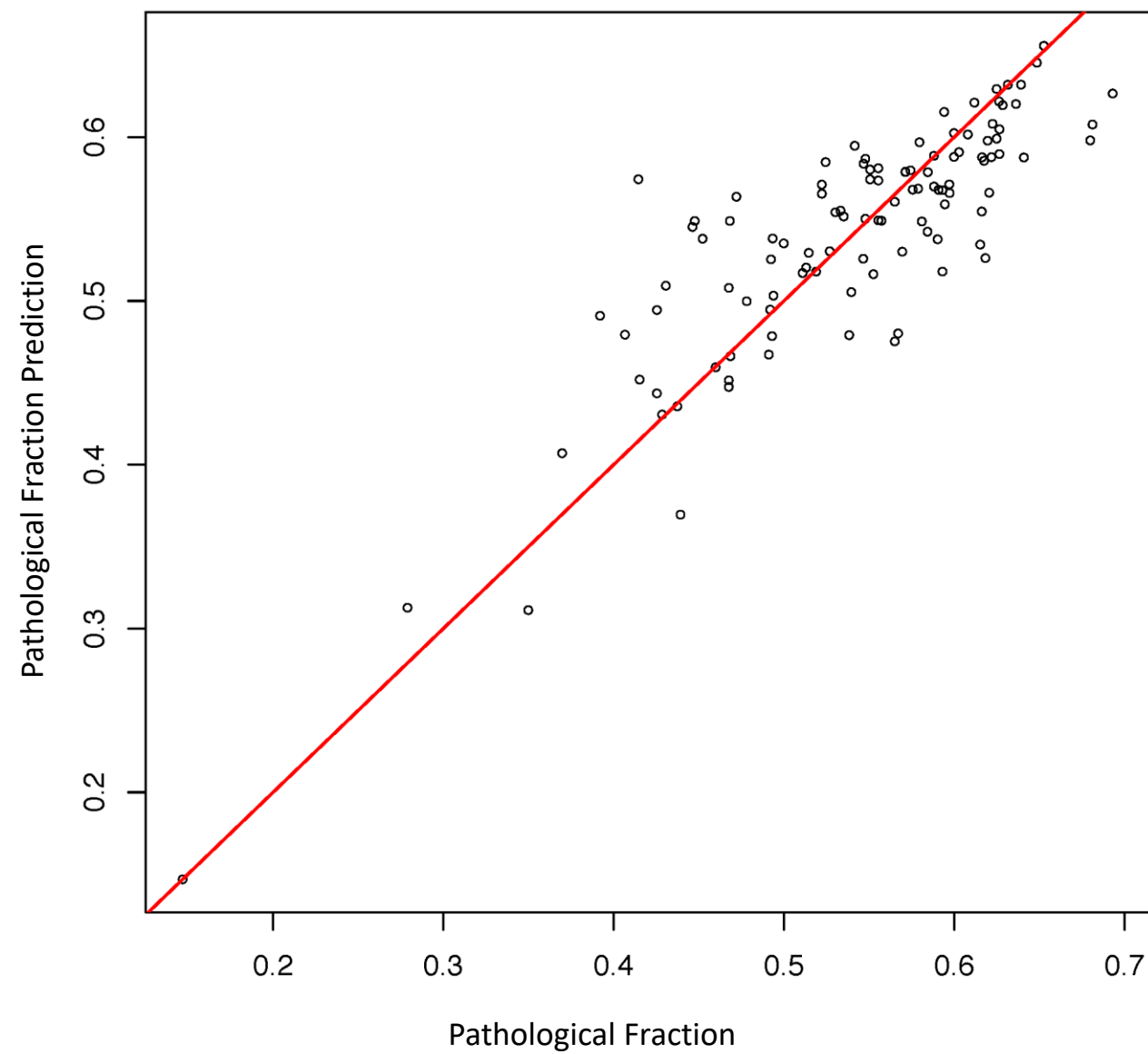

Supplement: Supplementary file 1 [file biomedicines-10-02044-s001.zip › Figure S1.pdf]
